# Clinical and Histological Predictors of Advanced Basal Cell Carcinoma Recurrence After Complete Response to Hedgehog Pathway Inhibitors: A Retrospective Multicenter Observational Study

**DOI:** 10.3390/cancers17111840

**Published:** 2025-05-30

**Authors:** Maria Mannino, Massimiliano Scalvenzi, Alessandro Di Stefani, Claudia Costa, Piergiacomo Calzavara-Pinton, Maria Concetta Fargnoli, Alfredo Piccerillo, Enrico Bocchino, Iris Zalaudek, Paolo Antonio Ascierto, Pietro Quaglino, Paola Queirolo, Emi Dika, Vincenzo De Giorgi, Elvira Moscarella, Luca Bianchi, Caterina Longo, Carmen Cantisani, Alessia Villani, Ketty Peris, Collaborators

**Affiliations:** 1 UOC di Dermatologia, Dipartimento di Scienze Mediche e Chirurgiche Addominali ed Endrocrino Metaboliche, Fondazione Policlinico Universitario A. Gemelli—IRCCS, 00168 Rome, Italy; mariamannino04@yahoo.it (M.M.);; 2 Dermatologia, Università Cattolica del Sacro Cuore, 00168 Rome, Italy; 3 Section of Dermatology, Department of Clinical Medicine and Surgery, University of Naples Federico II, 80125 Naples, Italy; 4 Department of Dermatology, University of Brescia, 25121 Brescia, Italy; 5 Department of Biotechnological and Applied Clinical Sciences, University of L’Aquila, 67100 L’Aquila, Italy; 6 Department of Dermatology and Venerology, University of Trieste, 34127 Trieste, Italy; 7 Istituto Nazionale Tumori IRCCS Fondazione “G. Pascale”, 80131 Napoli, Italy; 8 Dermatologic Clinic, Department of Medical Sciences, University of Turin, 10124 Torino, Italy; 9 Division of Medical Oncology for Melanoma, Sarcoma, and Rare Tumors, European Institute of Oncology IRCCS, 20141 Milan, Italy; 10 Oncologic Dermatology Unit, IRCCS Azienda Ospedaliero Universitaria di Bologna, 40138 Bologna, Italy; 11 Department of Medical and Surgical Sciences, University of Bologna, 40126 Bologna, Italy; 12 Dermatology, Department of Health Sciences, University of Florence, 50121 Florence, Italy; 13 Dermatology Unit, University of Campania “Luigi Vanvitelli”, 80131 Naples, Italy; 14 Dermatology Unit, Policlinico Tor Vergata, 00133 Rome, Italy; 15 Department of Dermatology, University of Modena and Reggio Emilia, 41121 Modena, Italy; 16 Skin Cancer Center, Azienda Unità Sanitaria Locale—IRCCS di Reggio Emilia, 42122 Reggio Emilia, Italy; 17 Department of Dermatology, University of Rome La Sapienza, 00185 Rome, Italy

**Keywords:** advanced basal cell carcinoma, non-melanoma skin cancer, hedgehog pathway inhibitors, sonidegib, systemic therapy, tumor recurrence, long-term follow-up

## Abstract

*Background*/*Objectives*: Long-term outcome data of locally advanced basal cell carcinoma patients who achieve complete response (CR) on hedgehog pathway inhibitors (HHIs) are lacking, highlighting a gap in the identification of the predictors of tumor recurrence. We aimed to investigate the clinical and histological factors associated with locally advanced BCC recurrence after CR on HHIs. *Patients and methods*: We performed a retrospective multicenter observational study at 14 Italian tertiary referral centers (1 January 2016–1 March 2024). Univariate logistic regression was used to investigate the association between locally advanced BCC recurrence and clinical and histological features. Kaplan–Meier analysis was used to estimate relapse-free survival (RFS). *Results*: A total of 106 locally advanced BCC patients were enrolled, of whom 14/106 (13.2%) experienced relapse after a median follow-up of 12 months (range: 1–70 months). Low-risk locally advanced BCC histological subtypes (superficial and nodular) were associated with a decreased probability of locally advanced BCC recurrence (odds ratio [OR]: 0.15, 95% CI: 0.04–0.51; *p*: 0.003); a longer time to CR predicted locally advanced BCC relapse (OR: 1.07, 95% CI: 1.01–1.15; *p*: 0.04). Accordingly, locally advanced BCC histology and time to CR significantly impacted RFS probability. *Conclusions*: Specific locally advanced BCC histological subtypes and time to CR predict tumor recurrence after CR on HHIs.

## Simple Summary

Data on the long-term outcomes of advanced basal cell carcinoma patients who achieve complete response (CR) on sonidegib or vismodegib are scarce, highlighting an unmet need concerning the identification of possible predictors of tumor recurrence after CR achievement. Our aim was to investigate the clinical and histological predictors of advanced BCC recurrence in a cohort of locally advanced BCC patients who achieved CR on sonidegib/vismodegib at 14 Italian tertiary referral centers. We enrolled 106 locally advanced BCC patients, of whom, after a median follow-up of 12 months (range: 1–70 months), 14/106 patients (13.2%) recurred after CR achievement. High-risk histological subtypes and longer time to CR were significantly associated with an increased risk of relapse and reduced probability of relapse-free survival.

## 1. Introduction

Basal cell carcinoma (BCC) is the most common malignancy in the white population, and it accounts for 75% of all skin cancers [1]. The majority of primary BCCs can be successfully treated by surgery and radiotherapy (RT), as well as topical and destructive treatments for low-risk superficial BCC subtypes [2].

Advanced BCC accounts for less than 1% of all BCC diagnoses, and it includes locally advanced BCC and metastatic BCC, for which curative surgery or RT is not feasible due to tumor and/or patient related factors [2,3]. Systemic therapy with hedgehog pathway inhibitors (HHIs) represents the standard of care for advanced BCC patients [4,5]. Vismodegib was the first-in-class smoothened (SMO) inhibitor, and it was granted Food and Drug Administration (FDA) and European Medicines Agency (EMA) approval for locally advanced and metastatic BCC [6]. Sonidegib was the second SMO inhibitor to be introduced on the market, and it is approved for locally advanced BCC patients [7], as well as metastatic BCC in Switzerland and Australia.

The pivotal phase II ERIVANCE and BOLT trials investigated the efficacy and safety profile of vismodegib and sonidegib, respectively [4,5]. Despite the differences in the efficacy assessment criteria in the ERIVANCE and BOLT trials, vismodegib and sonidegib share a similar efficacy profile [8]: the 39-month investigator-assessed objective response rate (ORR) was 60.3% for locally advanced BCC patients treated with vismodegib [9], and the 42-month central review-assessed ORR was 56% for locally advanced BCC patients on sonidegib [10]. Safety analyses have demonstrated the great frequency of treatment emergent adverse events in almost all advanced BCC patients, with approximately one-third of the trial population experiencing grade 3 and 4 toxicities [9,10].

Data about the long-term outcomes of advanced BCC patients who achieve complete response (CR) on HHIs are scarce [11], highlighting a major unresolved clinical question on the identification of relevant predictors of tumor recurrence after CR. The recognition of a patient population more prone to disease relapse, and for whom a tailored follow-up approach or continuing HHI treatment beyond CR might be beneficial, is likely to improve the long-term management of advanced BCC patients.

The aim of the present study was to investigate the clinical and histological characteristics associated with locally advanced BCC recurrence after CR on HHI.

## 2. Patients and Methods

### 2.1. Study Design and Data Collection

We performed a retrospective multicenter observational study at fourteen Italian tertiary referral centers in the period 1 January 2016–1 March 2024. Inclusion criteria were as follows: adult patients with histologically confirmed locally advanced BCC diagnosis who achieved CR on HHI treatment (vismodegib or sonidegib). Patients were deemed to have locally advanced BCC as per the decision of the local multidisciplinary tumor board of each enrolling tertiary referral center, according to the European guidelines’ recommendations [2]. CR was defined as the absence of clinical and dermoscopic signs of the target locally advanced BCC lesion, or the absence of residual neoplastic cells on histology.

The following clinical data were collected: patient’s age and sex, Eastern Cooperative Oncology Group Performance Status (ECOG PS), locally advanced BCC histological subtype (superficial, nodular, morpheiform, basosquamous, infiltrative, and micronodular), primary tumor site, BCC treatments prior to HHI therapy, the date of HHI start and discontinuation, and the time of CR assessment and of tumor recurrence. For locally advanced BCC histological subtype classification, single or multiple incisional biopsies of the target BCC lesion were performed, as per the decision of each enrolling center. In the case of the diagnosis of two different histological subtypes (e.g., low-risk histology, superficial and nodular BCC; high-risk histology, morpheiform, basosquamous, infiltrative, and micronodular), patients were classified as the high-risk histological subtype. The methods used to investigate tumor recurrence included clinical and dermatoscopic examination and histology. BCC recurrence was defined as the clinical, dermatoscopic, and histological evidence of tumor reappearance at the site of the primary locally advanced BCC lesion. Time to CR was defined as the time from the first dose of HHIs until CR assessment; time to tumor recurrence was calculated as the time from the first documentation of CR until locally advanced BCC relapse. Relapse-free survival (RFS) was calculated as the time from CR assessment until locally advanced BCC relapse or patient death. Patients who did not experience tumor recurrence or death were censored at the date of the last follow-up visit.

### 2.2. Statistical Analysis

Continuous variables were reported as median and range; categorical variables were summarized as numbers and percentages. We applied Fisher’s exact test and the Mann–Whitney test to detect differences between relapsing and non-relapsing locally advanced BCC patient cohorts. Univariate logistic regression was used to investigate the association between locally advanced BCC recurrence after CR and clinical and histological features; results were presented as odds ratio (OR) and 95% confidence interval (CI). Kaplan–Meier analysis was used to estimate RFS, and the log-rank test to detect differences between the curves. A *p* value < 0.05 was chosen as threshold level of statistical significance. All statistical analyses were performed with GraphPad Prism, version 10.0.

## 3. Results

### 3.1. Patient Population

We included 106 locally advanced BCC patients who achieved CR on HHIs, out of a total of 199 locally advanced BCC patients who had been treated with HHIs at fourteen Italian tertiary referral centers (a subset of these locally advanced BCC patients on HHI treatment has already been described elsewhere [12]). The median age of our cohort was 78 years (range: 28–97 years), with males accounting for 64.2% (n = 68) of the patient population (Table 1). BCC lesions were mostly located in the head and neck region (n = 79, 74.5%), followed by the trunk and upper and lower extremities (n = 27, 25.5%). Histological subtypes included superficial and nodular BCCs (low-risk histology) in 66% (n = 70) of cases, whereas high-risk histological subtypes, including morpheiform, basosquamous, infiltrative, and micronodular BCCs, accounted for 44% (n = 36) of the locally advanced BCC diagnoses. Ninety-four patients (88.7%) were treated with sonidegib, and twelve (11.3%) with vismodegib. Median HHI therapy duration was 18 months (range: 1–37 months), median time to CR was 8 months (range: 1–34 months), and the median duration of HHI treatment beyond CR achievement was 6 months (range: 0–22 months) (Table 1).

After a median follow-up of 12 months (range: 1–70 months), 14/106 patients (13.2%) experienced BCC relapse after CR achievement on HHIs (vismodegib n = 5; sonidegib n = 9), of whom 10 (71.4%) continued HHI treatment beyond CR (vismodegib n = 3; sonidegib n = 7). We reported a significant difference in the locally advanced BCC histological subtypes between relapsing and non-relapsing patients, with a higher percentage of high-risk histological subtypes (morpheiform, basosquamous, infiltrative, and micronodular) in the relapsing cohort (71.4%) versus the non-relapsing cohort (28.3%), *p* value: 0.004 (Table 1). The median time to achieve CR was significantly longer in recurring locally advanced BCCs (12.5 months [range: 5–39 months]) versus the non-recurring group (7 months [range: 1–32 months], *p*: 0.04). A nearly significant value was reported comparing the median duration of HHI treatment between locally advanced BCC patients who experienced tumor relapse (15 months [range: 7–20 months]) versus locally advanced BCC patients who did not recur (18 months [range: 1–37 months], *p*: 0.06). Likewise, we found a tendency towards a difference between relapsing and non-relapsing patients with regard to the time on HHI treatment beyond CR achievement, *p*: 0.14 (4.5 months [range: 0–16 months] and 6 months [range: 0–22 months], respectively) (Table 1).

### 3.2. Clinical and Histological Features Associated with Locally Advanced BCC Recurrence After CR

Low-risk locally advanced BCC histological subtypes (superficial and nodular) were significantly associated with a decreased probability of tumor recurrence after CR on HHIs (OR: 0.15, 95% CI: 0.04–0.51; *p*: 0.003) (Table 2). Also, time to CR significantly predicted locally advanced BCC relapse after CR achievement (OR: 1.07, 95% CI: 1.01–1.15; *p*: 0.04), with an additional risk of 7% per every month increase in the time needed to reach tumor clearance. We found a nearly significant value with regard to the maintenance of HHI treatment beyond CR and the risk of relapse, with a trend towards a risk reduction of 9% per every additional month of HHI treatment continuation beyond CR achievement (OR: 0.91, 95% CI: 0.81–1.00; *p*: 0.07). Total HHI treatment duration was not significantly associated with a decline in the risk of locally advanced BCC recurrence after CR (*p*: 0.11, Table 2).

**Figure 1:**
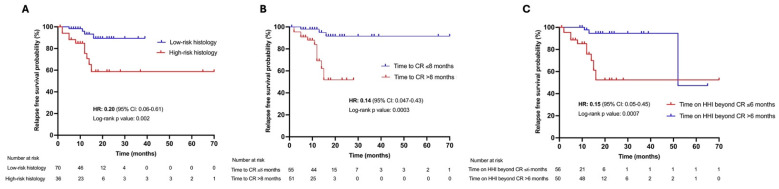
Relapse-free survival probability of patients grouped according to histological subtypes (low- versus high-risk histology) (**A**); median time to complete response achievement (**B**); median hedgehog pathway inhibitor treatment continuation beyond complete response (**C**). Abbreviations: CI, confidence interval; CR, complete response; HHI, hedgehog pathway inhibitor; HR, hazard ratio.

The RFS probability of our cohort was 77.8% by the end of the follow-up period (Supplementary Figure S1), and the median RFS was not reached. The RFS rate was 89.4% for low-risk locally advanced BCC histological subtypes (superficial and nodular); in line with the logistic regression results, low-risk locally advanced BCC histology was significantly associated with a higher RFS probability compared to high-risk histology (hazard ratio [HR]: 0.2, 95% CI: 0.06–0.61; *p*: 0.002) (Figure 1A). We investigated RFS rates according to the primary tumor site (head and neck localization versus trunk and limbs): despite not reaching a level of statistical significance (*p*: 0.08), lesions located in the head and neck area presented with a tendency towards a 60% reduction in the risk of tumor relapse after CR (HR: 0.40, 95% CI: 0.11–1.39) (Supplementary Figure S2). We found a higher RFS probability for patients who required ≤8 months to achieve CR after HHI start, compared to patients who needed >8 months from the first dose of HHI until tumor clearance (91.6% and 51.8%, respectively; HR: 0.14, 95% CI: 0.047–0.43; *p*: 0.0003) (Figure 1B). For patients who continued HHI treatment beyond CR and for >6 months, we reported an 85% risk reduction in relapse probability, versus patients who stopped or maintained HHI treatment after CR achievement for ≤6 months (HR: 0.15, 95% CI: 0.05–0.45; *p*: 0.0007) (Figure 1C).

## 4. Discussion

The present study investigated the clinical and histological features associated with locally advanced BCC recurrence after CR achievement on vismodegib or sonidegib treatment. We identified histological subtypes (low-risk versus high-risk histology) and time to CR achievement as relevant predictors of tumor recurrence; accordingly, these factors along with the continuation of sonidegib or vismodegib treatment beyond tumor clearance significantly impacted the RFS probability of the locally advanced BCC patient population.

In our cohort, 13.2% of patients (14/106) experienced tumor relapse after a median follow-up of 12 months. This percentage is considerably smaller compared to the results reported by Herms et al. [13] and Bassompierre et al. [14], who observed a relapse rate of 46.6% after a median follow-up of 34.9 months and of 48.1% after a median follow-up of 21 months, respectively (Supplementary Table S1). Our patient population underwent a median follow-up of 12 months only, with a limited number of patients reaching the end of the follow-up period. Also, a significant proportion of our cohort (n = 73, 68.9%) continued HHI treatment beyond CR achievement, whereas in two French retrospective studies, patients discontinued vismodegib upon or shortly after aBCC clearance (median time of 1 month). Altogether, these factors may explain our limited tumor relapse rate. The maintenance of HHI therapy beyond CR achievement significantly affected the RFS rates, with a risk reduction for patients continuing HHI > 6 months compared to patients who stopped or underwent treatment duration for ≤6 months after CR. An Italian multicenter retrospective study on vismodegib continuation after clinical CR achievement in advanced BCC patients reported a similar result, with longer disease-free survival rates for patients who continued HHI treatment beyond CR (Supplementary Table S1) [15]. Another retrospective observational study by Scalvenzi et al. [16] enrolled locally advanced BCC patients who reached tumor clearance on vismodegib treatment: for patients who continued low-dose vismodegib beyond CR (once-weekly vismodegib administration for 1 year after CR), no advanced BCC recurrences were observed during a 1-year follow-up period. HHIs lead to tumor regression via the inhibition of the proliferation and apoptosis of cancer cells [17]; nevertheless, a small subset of tumor cells is able to persist in a quiescent state, and it is responsible for driving BCC recurrence after treatment discontinuation [18]. Extending HHI treatment beyond CR achievement is likely to put a brake on locally advanced BCC relapses. Further prospective studies are needed to provide optimal guidance on the long-term management of advanced BCC patients after CR: the ongoing phase II open-label SONIBEC trial (NCT04806646) is expected to clarify the unmet need of a tailored HHI schedule after CR, maximizing treatment efficacy and balancing tolerability.

In the present study, longer time to CR achievement and high-risk advanced BCC histology significantly predicted tumor recurrence: it is possible that patients with these tumor characteristics present with a biologically more aggressive disease subset warranting a short follow-up schedule. Previous studies [12,19,20] reported an association between high-risk BCC histological subtypes (morpheiform, basosquamous, infiltrative, and micronodular) and disease progression on HHI treatment, highlighting the complexity of treatment success and higher risk of relapse for this patient group. With regard to the basosquamous BCC histology, it has recently been described as a pre-existing and HHI-resistant cellular population that is marked with the surface antigen LYD6, and it is likely to drive the poor clinical outcomes of this BCC subtype [21]. We reported a nearly significant figure with regard to the locally advanced BCC primary tumor site and the RFS rates of our cohort: patients presenting with locally advanced BCCs located in the head and neck area were less likely to experience tumor recurrence after CR. This finding is in line with the French retrospective study by Herms et al. [13], which found a greater relapse rate for advanced BCCs arising in areas different from the head and neck localization. Chronic ultraviolet (UV) light exposure is less prominent for advanced BCCs located on the trunk and limbs; BCCs on different body sites might display a different molecular signature that could possibly affect the RFS rates after tumor clearance on HHIs.

The assessment and grading of treatment emergent adverse events was beyond the scope of the present manuscript, as was the investigation of the reasons for sonidegib or vismodegib discontinuation and the implementation of subsequent lines of therapy in case of advanced BCC recurrence, such as HHI rechallenge, anti-programmed-death-1 (PD-1) immunotherapy, surgery, or RT. We acknowledge that these factors, as well as the retrospective nature of our multicenter study, are limitations.

## 5. Conclusions

In conclusion, our study provides further insights into the identification of predictive factors of locally advanced BCC recurrence after CR achievement on sonidegib or vismodegib treatment. Patients requiring longer time to CR and presenting with high-risk BCC histological subtypes are likely to represent a more aggressive BCC population which warrants a tailored long-term management; the continuation of HHI treatment beyond CR achievement positively affects the RFS rates of locally advanced BCC patients.

## Figures and Tables

**Table 1:** Clinical and histological characteristics of recurring and non-recurring locally advanced basal cell carcinoma patients.

Variable	Overall Cohort (N = 106)	Tumor Recurrence After CR, Yes (N = 14)	Tumor Recurrence After CR, No (N = 92)	*p* Value
**Age (years), median (range)**	78 (28–97)	77 (51–97)	78 (28–96)	0.73
**Gender, N (%)**				
Male	68 (64.2)	8 (57.1)	60 (65.2)	0.56
Female	38 (35.8)	6 (42.9)	32 (34.8)	
**ECOG PS, N (%)**				
0–1	101 (95.3)	12 (85.7)	89 (96.7)	0.12
≥2	5 (4.7)	2 (14.3)	3 (3.3)	
**Immunosuppression, N (%)**				
Yes	12 (11.3)	3 (21.4)	9 (9.8)	0.19
No	94 (88.7)	11 (78.6)	83 (90.2)	
**LaBCC site, N (%)**				
Head and neck	79 (74.5)	8 (57.1)	71 (77.2)	0.18
Trunk and limbs	27 (25.5)	6 (42.9)	21 (22.8)	
**LaBCC histology, N (%)**				
High risk *	36 (44)	10 (71.4)	26 (28.3)	**0.004**
Low risk ^±^	70 (66)	4 (28.6)	66 (71.7)	
**Therapies prior to HHIs, N (%)**				
Yes	60 (56.6)	9 (64.3)	51 (55.4)	0.57
No	46 (43.4)	5 (35.7)	41 (44.6)	
**HHI duration (months), median (range)**	18 (1–37)	15 (7–20)	18 (1–37)	0.06
**Time to CR (months), median (range)**	8 (1–34)	12.5 (5–39)	7 (1–32)	**0.04**
**HHIs beyond CR, N (%)**				
Yes	73 (68.9)	10 (71.4)	63 (68.5)	0.90
No	33 (31.2)	4 (28.6)	29 (31.5)	
**HHI duration (months), median (range)**	6 (0–22)	4.5 (0–16)	6 (0–22)	0.14

Abbreviations: laBCC, locally advanced basal cell carcinoma; CR, complete response; ECOG PS, Eastern Cooperative Oncology Group Performance Status; HHI, hedgehog inhibitor therapy; N, number; *: morpheiform, basosquamous, infiltrative, and micronodular; ^±^: superficial and nodular.

**Table 2:** Clinical and histological predictors of locally advanced basal cell carcinoma recurrence after complete response.

Variable	Outcome: Tumor Recurrence After CR		*p* Value
	OR	95% CI	
**Age (years)**	0.99	0.95–1.04	0.73
**Gender**			
Female	1.40	0.42–4.39	0.55
Male			
**ECOG PS**	1.35	0.50–3.29	0.51
**LaBCC location**			
Head and neck	0.11	0.76–8.13	0.11
Trunk and limbs			
**LaBCC histology**			
High risk *	0.15	0.04–0.51	**0.003**
Low risk ^±^			
**Therapies prior to HHI**			
Yes	0.69	0.19–2.16	0.53
No			
**HHI duration (months)**	0.94	0.86–1.01	0.11
**Time to CR (months)**	1.07	1.01–1.15	**0.04**
**Time on HHI beyond CR (months)**	0.91	0.81–1.00	0.07

Abbreviations: laBCC, locally advanced basal cell carcinoma; CI, confidence interval; CR, complete response; ECOG PS, Eastern Cooperative Oncology Group Performance Status; HHI, hedgehog inhibitor therapy; N, number; OR, odds ratio; *: morpheiform, basosquamous, infiltrative, and micronodular; ^±^: superficial and nodular.

## Data Availability

The data that support the findings of this study are available on request from the corresponding author, Ketty Peris.

## Supplementary Materials

The following supporting information can be downloaded at https://www.mdpi.com/article/10.3390/cancers17111840/s1, Figure S1: Relapse-free survival probability of the overall cohort; Figure S2: Relapse-free survival probability of patients grouped according to tumor site; Table S1. Summary of studies investigating advanced BCC relapse after CR on HHIs.
