# Supplementary material for: Clinical and Histological Predictors of Advanced Basal Cell Carcinoma Recurrence After Complete Response to Hedgehog Pathway Inhibitors: A Retrospective Multicenter Observational Study
Source: Cancers (Basel). 2025 May 30;17(11):1840. doi: 10.3390/cancers17111840 (PMC12153653; doi:10.3390/cancers17111840)
Supplement: Supplementary file 1 [file cancers-17-01840-s001.zip › cancers-3613522-supplementary.pdf]

Supplementary Figures and Tables

Supplementary Figures

Supplementary Figure S1. Relapse-free-survival probability of the overall cohort

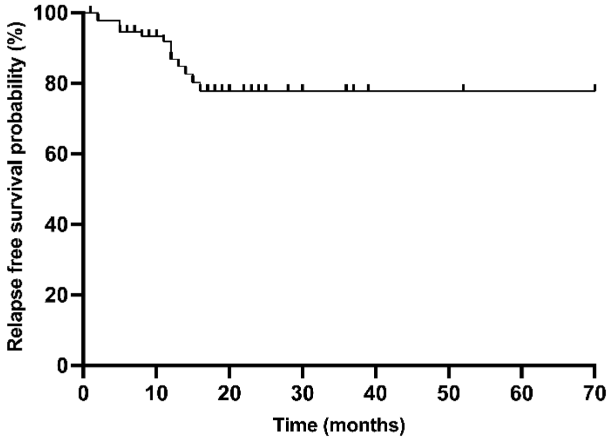

Number at risk	106	69	18	7	3	3	2	1

Supplementary Figure S2. Relapse-free-survival probability of patients grouped according to tumor site

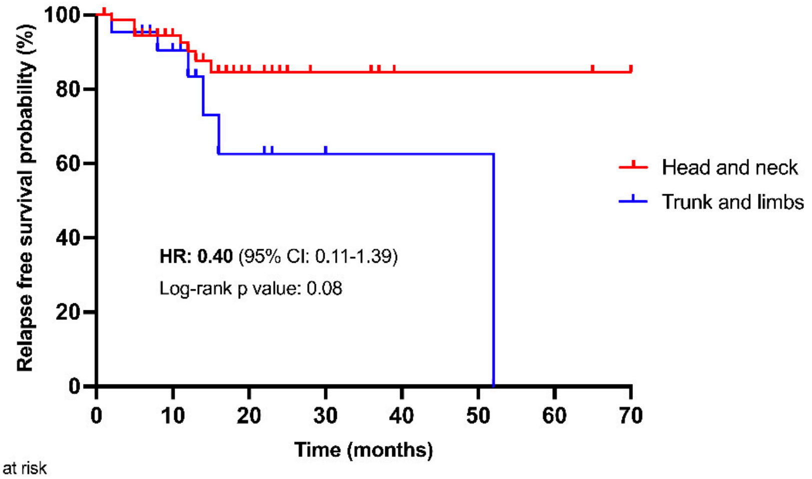

Number at risk								
Head and neck	79	52	14	4	2	2	2	1
Other	27	17	4	3	1	1	0	0

Supplementary Tables

Supplementary Table S1. summary of studies investigating advanced BCC relapse after CR on HHI

Study reference	Study design	Number of patients	Median FUP	Median RFS/DFS
Herms F. et al. [13]	Multicenter, observational, retrospective<br><br>LaBCC with CR on vismodegib who discontinued treatment	116	34.9 months	RFS: 18.4 months (95% CI: 13.5-24.8 months)
Bassompiere A. et al. [14]	Multicenter, observational, retrospective<br><br>LaBCC and mBCC with CR on vismodegib who discontinued treatment	110	21 months	RFS: 24 months (IQR: 13.0-38.0 months)
Alfieri S. et al. [15]	Multicenter, observational, retrospective<br><br>LaBCC with CR on vismodegib who discontinued or continued treatment beyond CR	68<br><br>61/68 stopped vismodegib<br>7/68 continued vismodegib	42.5 months	Vismodegib discontinuation, DFS: 365.0 days (range: 0-2600 days)<br>Vismodegib beyond CR, DFS: 1261.0 days (range: 182-1955 days)
Scalvenzi M. et al. [16]	Single center, observational, retrospective<br><br>LaBCC with CR on vismodegib who discontinued or continued treatment ("low-dose") beyond CR	42<br><br>15/42 stopped vismodegib<br>27/42 continued vismodegib	NA	NA

Abbreviations: CI, confidence interval; CR, complete response; DFS, disease free survival; FUP, follow-up; IQR, interquartile range; LaBCC, locally advanced basal cell carcinoma; mBCC, metastatic basal cell carcinoma; NA, not available; RFS, relapse free survival
